# Supplementary material for: Targeting a critical step in fungal hexosamine biosynthesis
Source: J Biol Chem. 2020 Apr 27;295(26):8678–91. doi: 10.1074/jbc.RA120.012985 (PMC7324522; doi:10.1074/jbc.RA120.012985)
Supplement: Supporting Information [file supp_295_26_8678__index.html]

Targeting a critical step in fungal hexosamine biosynthesis — Targeting a critical step in fungal hexosamine biosynthesis — Supporting Information 

# Targeting a critical step in fungal hexosamine biosynthesis

## Supporting Information

- Supporting Information (to be published online) - Supporting Information
